# Durability of Immune Response after Application of a Third Dose of SARS-CoV-2 Vaccination in Liver Transplant Recipients

**DOI:** 10.3390/vaccines11030572

**Published:** 2023-03-02

**Authors:** Moritz Passenberg, Roxane Authorsen-Grudmann, Alexandra Frey, Johannes Korth, Jaqueline Zmudzinski, Olympia E. Anastasiou, Birte Möhlendick, Hartmut Schmidt, Jassin Rashidi-Alavijeh, Katharina Willuweit

**Affiliations:** 1 Department of Gastroenterology, Hepatology and Transplantation Medicine, University Hospital Essen, University of Duisburg-Essen, Hufelandstr. 55, 45147 Essen, Germany; 2 Department of Nephrology, University Hospital Essen, University of Duisburg-Essen, Hufelandstr. 55, 45147 Essen, Germany; 3 Institute for Virology, University Hospital Essen, University of Duisburg-Essen, Virchowstr. 179, 45147 Essen, Germany; 4 Institute of Pharmacogenetics, University Hospital Essen, University of Duisburg-Essen, Hufelandstr. 55, 45147 Essen, Germany

**Keywords:** SARS-CoV-2, vaccination, liver transplant recipients, liver transplantation, COVID-19

## Abstract

Immunogenicity after SARS-CoV-2 vaccination is known to be impaired in liver transplant (LT) recipients, but the results after the application of a third dose show significant improvement in seroconversion rates. In the general population, the antibody response wanes over the course of time after two doses of the vaccination, but seems to be more robust after the application of three doses. Still, the durability of the antibody response in LT recipients who receive a third dose of SARS-CoV-2 vaccination has not been analyzed yet. We therefore assessed antibody responses in a total of 300 LT recipients and observed antibody titers for six months each after patients had received the second and the third doses of the vaccination, explicitly excluding all patients who had suffered from SARS-CoV-2 infection. The initial antibody response was compared to a control group of 122 healthcare workers. After the application of two doses of the vaccination, 74% of LT recipients (158 out of 213) developed antibodies against SARS-CoV-2; this result depended significantly on whether the patients were taking the medication mycophenolate mofetil, and on the age of the patients. Antibody titers declined significantly within six months from 407 BAU/mL (IQR: 0–1865) to 105 BAU/mL (IQR: 0–145) (*p* ≤ 0.001), but increased after the application of the third vaccine dose in 92% of patients (105 out of 114), showing an antibody response (*p* ≤ 0.001). After a further six-month period, despite showing a decline from 2055 BAU/mL (IQR: 500 to >2080) to 1805 BAU/mL (IQR: 517 to >2080), the waning of antibody titers was not significant (*p* = 0.706), and antibody durability appeared to be more robust than that after the second dose. In conclusion, our study confirms the high efficacy of the application of a third dose of SARS-CoV-2 vaccination in LT recipients, and a reasonably sustained humoral response with superior durability in comparison to antibody kinetics after the application of the second dose of the vaccination.

## 1. Introduction

In comparison to other solid organ transplant (SOT) recipients [1], vaccination against SARS-CoV-2 infection is known to generate superior humoral responses in liver transplant (LT) recipients after two doses of the mRNA-based vaccine BNT162b2 [2,3], resulting in an efficient reduction in mortality after SARS-CoV-2 infection [4]. However, antibody responses are still impaired in LT recipients, with the most significant reduction in antibody titers occurring in patients of older age or those receiving mycophenolate mofetil as an immunosuppressive medication [5,6]. Considering the probable severe course of SARS-CoV-2 infection in immunocompromised patients, optimizing their immune response is of crucial importance. In this regard, different groups reported their experiences after the application of a third dose of the SARS-CoV-2 vaccine in SOT and LT recipients, with encouraging initial immune responses [7,8,9,10,11,12].

Despite these positive initial results, the data on the durability of antibody responses in LT recipients are limited. Still, this information is of vital importance, since it can help to find the optimal time for a potential fourth vaccination dose. The waning of antibody responses over the course of time after two doses of the SARS-CoV-2 vaccine was described in the general population [13,14] and in LT recipients [15]. Current data suggest a more robust antibody response in the general population after three doses of the SARS-CoV-2 vaccine [16]. However, Kamar et al. reported a significant decline in antibody response in SOT recipients three months after receiving the third dose of the SARS-CoV-2 vaccine [17]. Still, data on the longevity of the antibody response in LT recipients after receiving three doses of the SARS-CoV-2 vaccination are lacking.

For the above-mentioned reasons, in our study, we assess the immunogenicity of a third dose of the SARS-CoV-2 vaccine in LT recipients, and also focus on the durability of the antibody response.

## 2. Materials and Methods

In total, 300 liver transplant recipients and 122 healthcare workers (HCW) were enrolled in this study. Both LT recipients and HCWs who had developed SARS-CoV-2 infection before vaccination, patients aged under 18, and patients who were pregnant were excluded from this analysis. There was no serological screening before the initial vaccination, and SARS-CoV-2 infection prior to vaccination was only excluded via self-report or if the patients had been tested due to symptoms. In addition, patients were excluded from further testing if they had SARS-CoV-2 infection during the course of observation. All patients and HCWs received the mRNA-based SARS-CoV-2 vaccine BNT162b2 (Pfizer-BioNTech, Pfizer Inc., New York City, NY, USA and BioNTech SE, Mainz, Germany) according to the standard protocol for the first and second vaccination. For the third vaccination, patients received either the vaccine BNT162b2 or mRNA-1273 (Spikevax^®^ (Moderna), ModernaTX Inc.; Cambridge, MA, USA). Serum samples were tested for SARS-CoV-2 IgG against the spike glycoprotein using a validated anti-SARS-CoV-2 IgG assay (LIAISON^®^ SARS-CoV-2 TrimericS IgG assay, Diasorin, Saluggia, Italy). A ratio of <13.0 arbitrary units per milliliter (AU/mL) was defined to be negative and a ratio of ≥13 AU/mL to be positive, according to the manufacturer’s recommendations. To convert the arbitrary units per milliliter to binding antibody units (BAU/mL) that correlate with the WHO standard, the following equation was used: 2.6* AU/mL = BAU/mL. Here, 800.0 AU/mL (2080 BAU/mL) is the upper limit of quantification without dilution. Serum samples were tested at different time points; initial testing was performed in a median of 15 days (IQR: 14–24) for LT recipients and a median of 34 days (IQR: 33–36) for HCW after the second intramuscular vaccination. For further examination of the course of antibody titers over time, serum samples were tested again six months after the second vaccination, one month after third vaccination and again six months after third vaccination. Antibody titers over time were measured in LT recipients, but not in HCWs. It is important to note that tests were not paired at different time points; for this reason, 300 LT recipients was the total number of LT recipients included in the study, although the number of patients for each time was smaller.

Quantitative variables are given as median and interquartile range (IQR). Fisher’s exact test was used to compare categorical variables, and Mann–Whitney U and Kruskal–Wallis tests were used to compare continuous variables. A *p*-value < 0.05 was considered statistically significant. Statistical analysis was performed using SPSS 28 statistical software (IBM SPSS Statistics; IBM Corporation, Chicago, IL, USA). This study was conducted according to the guidelines of the Declaration of Helsinki and approved by the ethics committee of the Medical Faculty of the University of Duisburg-Essen (20-9753-BO).

## 3. Results

### 3.1. Baseline Characteristics

In total, 300 LT recipients who were followed up by our outpatient clinic for transplant were enrolled in our study. Of these, 167 (55.7%) were male and 133 (44.3%) were female. The median age at first vaccination was 56.5 years (IQR: 37–55) and the median time between LT and vaccination was 9 years (IQR: 5–14). The median time between the first and second vaccinations was 42 days (IQR: 35–42), and the median time between the second and third vaccinations was 187 days (IQR: 174–198) (Table 1). The most common indications for LT were alcohol-induced liver cirrhosis (*n* = 45, 15%) and hepatocellular carcinoma (*n* = 42, 14%), followed by autoimmune hepatitis (*n* = 40, 13%) (Table 2).

At the time of vaccination, most LT recipients received tacrolimus-based immunosuppression (*n* = 271, 90%). Of these, most obtained a combination therapy composed of tacrolimus and everolimus (*n* = 102, 38%) or tacrolimus and mycophenolate mofetil (MMF) (*n* = 88, 32%). Tacrolimus monotherapy was administered to 81 recipients (30%). Further, 20 patients (7%) received a cyclosporine A-based immunosuppression, and nine patients (3%) an everolimus-based immunosuppression. A total of 16 patients (7.5%) were receiving corticosteroid treatment at the time of vaccination, but none were receiving high-dose prednisolone.

### 3.2. Antibody Response and Titer after Second SARS-CoV-2 Vaccine Doses

**Figure 1:**
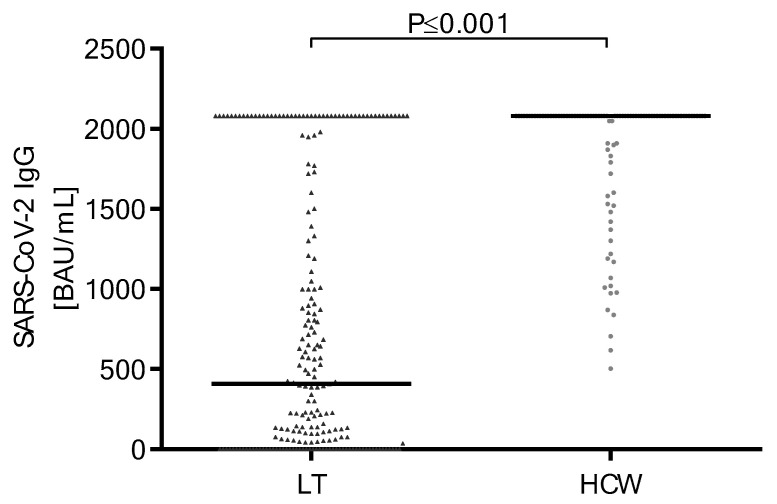
Comparison of the binding antibody units per milliliter (BAU/mL) ratio of SARS-CoV-2 IgG antibodies of LT recipients and HCWs after the second vaccination. LT: liver transplantation; HCWs: healthcare workers; BAU: binding antibody units; mL: milliliter; SARS-CoV-2: severe acute respiratory syndrome coronavirus type 2; IgG: immunoglobulin G.

Overall, 74% (*n* = 158 of 213) of LT recipients developed antibodies after a second dose of SARS-CoV-2 immunization, and 55 patients (26%) remained negative, whereas all HCWs (100%) developed an antibody response. Regarding antibody titers, LT recipients showed significantly lower antibody titers than the control group (407 BAU/mL (IQR: 0–1865) vs. greater than 2080 BAU/mL (IQR: 1878 to >2080) (*p* ≤ 0.001)) (Figure 1).

The comparison of seropositive and seronegative LT patients revealed no significant differences regarding sex (*p* = 0.210) or time between first and second vaccinations (*p* = 0.363). With regard to age, patients with seronegative antibody status were significantly older than patients with detectable antibodies after immunization (*p* = 0.015). Regarding immunosuppression, LT recipients who received MMF were less likely to develop antibodies compared to patients who did not receive MMF (*p* = 0.001). The administration of tacrolimus (*p* = 1.000), everolimus (*p* = 0.698), and prednisolone (*p* = 0.133) showed no significant impact on the immunogenicity of the vaccination (Table 3), nor did different combinations of immunosuppressive medication (data not shown).

### 3.3. Course of Antibody Titers after Second and Third SARS-CoV-2 Vaccinations in LT Recipients

As mentioned before, 158 (74%) of all LT recipients showed immunogenicity after the second vaccination, with a median antibody titer of 407 BAU/mL (IQR: 0–1865). Over the course of time, the antibody titers declined significantly, reaching a median titer of 105 BAU/mL (IQR: 0–451) six months after the second vaccination (*p* ≤ 0.001).

**Figure 2:**
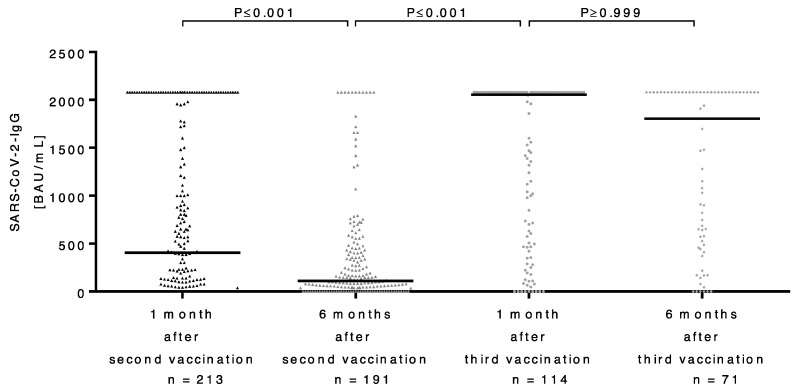
Course of binding antibody units per milliliter (BAU/mL) ratio of SARS-CoV-2 IgG antibody titers in LT patients after second and third vaccinations, as well as six months after. BAU: binding antibody units; mL: milliliter; SARS-CoV-2: severe acute respiratory syndrome coronavirus type 2; IgG: immunoglobulin G.

After the third vaccination, there was a significant improvement in immune responses, with 92% of patients developing antibodies and only 8% remaining negative. Antibody titers showed a significant increase, with a median titer of 2055 BAU/mL (IQR: 500 to >2080) one month after the third vaccination (*p* ≤ 0.001). After an additional six months following administration of the third vaccine, the antibody titers declined again, reaching a median antibody titer of 1805 BAU/mL (IQR: 517 to >2080). However, the decline in antibody titers six months after the third vaccination was not significant (*p* ≥ 0.999) (Figure 2). Still, the significance of this decline cannot be excluded since some of our patients had antibody titers above the upper limit of detection and no further dilution was performed.

### 3.4. Comparison of the Antibody Response in Patients with and without MMF after Application of Third Dose of SARS-CoV-2 Vaccine

**Figure 3:**
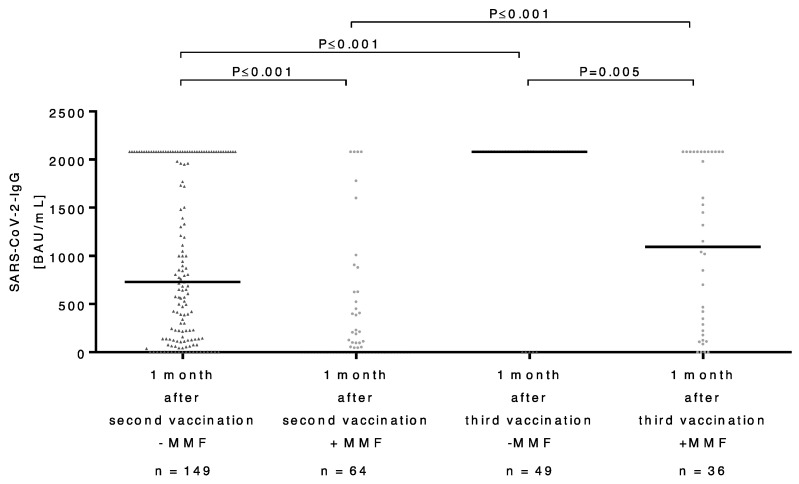
Comparison of the binding antibody units per milliliter (BAU/mL) ratio of SARS-CoV-2 IgG antibodies in LT patients with and without MMF administration one month each after the second and third vaccinations. BAU: binding antibody units; mL: milliliter; SARS-CoV-2: severe acute respiratory syndrome coronavirus type 2; IgG: immunoglobulin G; MMF: mycophenolate mofetil.

Since MMF administration resulted in a significantly impaired antibody response after the second vaccination, we compared the antibody titers of patients with and without MMF administration at different points in time after vaccination. Recipients without MMF had a median SARS-CoV-2 antibody level of 730 BAU/mL (IQR: 135 to >2080) after the second vaccination, whereas recipients with MMF administration had a significantly lower median SARS-CoV-2 antibody level (0 BAU/mL (IQR: 0–347); *p* ≤ 0.001). One month after the third vaccination, LT recipients both with and without the intake of MMF showed a significant increase in SARS-CoV-2 IgG antibody levels: patients who were not receiving MMF showed a median antibody titer of >2080 BAU/mL (IQR: 694 to >2080), while the median antibody titer in patients receiving MMF was 1095 BAU/mL (IQR: 109 to >2080). Although antibody titers after the third vaccination were significantly lower in patients who received MMF than in those who did not (*p* = 0.005), the third vaccination resulted in a significant increase, even in patients receiving MMF (*p* ≤ 0.001) (Figure 3).

## 4. Discussion

In this study we described the immunogenicity and durability of antibody response in a total of 300 LT recipients after two and three doses of the SARS-CoV-2 vaccine. After the administration of two vaccine doses, 74% of the LT recipients developed antibodies against SARS-CoV-2, with immunosuppressive therapy with MMF and advanced age being negatively associated with immunogenicity. Antibody titers declined significantly over the course of time, but showed an encouraging increase after the third dose, with 92% of patients showing an antibody response. As the main point of this study, the antibody titers were monitored for six months after the booster vaccination. Despite showing a decline, the waning of antibody titers was not significant, and antibody kinetics appeared to be more robust than the initial decrease after two doses of the vaccination; moreover, patients still showed relevant antibody titers six months after the application of the third vaccine.

The SARS-CoV-2 vaccination is known to induce slightly impaired, but still satisfactory antibody responses in patients with chronic liver disease [18,19,20]. In LT recipients, the antibody response is even more limited, although their immunogenicity is superior to those in other SOT recipients. In our cohort, we were able to confirm our previous findings, which are consistent with most previous studies reporting impaired immune response [2,3,5,6]. Aside from older age, immunosuppression with MMF was the main factor impairing the humoral response to vaccination. Medication with MMF not only impaired the initial antibody response after administration of the second dose of the SARS-CoV-2 vaccine, but also affected immunogenicity after the booster dose. This impact has already been observed in other studies, and the temporary pause of MMF application for the purpose of vaccine efficacy has already been discussed [21]. However, despite impairment of the immune response, patients with MMF still developed a significant increase in antibody titers after administration of their third dose, demonstrating the importance of repeated vaccination in this particular patient cohort.

The waning of antibody titers was observed over time after administration of the second dose of the SARS-CoV-2 vaccine. This decline was reported by several groups in the general population [13,14,22,23], as well as in LT recipients [7,15]. Although there is no clearly described threshold to define antibody titers that protect patients from severe courses of COVID-19, the levels of antibody titers are described to be associated with the degree of protection against infection, or at least against serious complications [24,25,26,27]. However, it must be mentioned that it is not entirely clear to what extent this statement can be applied to other SARS-CoV-2 variants [28]. In particular, Dimeglio et al. showed that there was a good correlation between antibody titers and infections for the Omicron BA.1 subvariant, whereas no such correlation was demonstrated for the Omicron BA.2 subvariant [29]. For this reason, differentiated consideration of the protective effect of the levels of antibody titers seems necessary. Therefore, the decline in antibody levels described in LT recipients within six months after vaccination raised serious concerns. In this context, results reported by different groups involving significant improvement in the humoral immune response after a third dose of the SARS-CoV-2 vaccination, and high rates of seroconversion, are encouraging [7,8,10,12]. We, too, observed a significant increase in immunogenicity after the third dose of the vaccination, with the rate of seroconversion increasing from 74% after two doses of the vaccination to 92% after three doses.

Still, little is known about the kinetics and durability of the antibody response after the third dose of the SARS-CoV-2 vaccination. A sustained humoral response has been reported in the general population after administration of the third vaccine dose, with longer persistence of antibodies compared to after the second dose [16]. However, antibody longevity in the context of immunosuppression may differ. In this context, Kamar et al. analyzed the durability of the antibody response in a large cohort of SOT recipients, and found that antibody responses waned significantly in a period of three months after patients received the third dose of the SARS-CoV-2 vaccination [17]. Still, LT recipients are known to show superior immunogenicity after two doses of SARS-CoV-2 vaccination in comparison to other SOT recipients, and consequently, there was hope of achieving superior durability after three doses of SARS-CoV-2 vaccination in these patients, too. Indeed, our results confirm reasonably robust antibody kinetics. The antibody response declined over the course of time, but appeared to decline to a lesser degree compared to the decline after the second dose, and patients still showed relevant antibody titers six months after the application of the third vaccine. The decline observed after the third vaccine was not significant, although it must be mentioned that the validity of this finding is limited, as some of the patients had a titer above the upper limit of detection; therefore, it was not possible to definitively clarify how strong the decline ultimately was.

Nonetheless, our study has its limitations, the most important being that our data concentrate on the measurement of antibody titers without further characterization of the immune response, such as analyzing the neutralizing capacity of the antibodies or T-cell activity. In addition, not all of our measurements were paired, and the absolute number of patients decreased over time, since many patients suffered from COVID-19 infection at some point in the study and were therefore excluded from further analysis. Moreover, serological screening before the initial vaccination of our patients or HCWs was not performed. For this reason, possible immune priming of undiagnosed pre-vaccination effects cannot be ruled out. The assay used only identifies SARS-CoV-2 IgG against spike glycoprotein, and differentiation between the vaccine response and pre-existing infection was therefore not possible; a combined assay also detecting SARS-CoV-2 nucleocapsid antibodies would be more useful [30,31]. Recently, our data concentrated on antibody titers and did not document breakthrough infections, which would have been of great interest to better understand correlations between antibody titers and the degree of protection.

Still, we are convinced that the results of our study have important clinical implications and are markedly meaningful for the vulnerable cohort of LT recipients. Our data confirm previous results and demonstrate satisfying antibody responses after a third dose of SARS-CoV-2 vaccination, with high rates of seroconversion. As a new finding, we report a reasonably sustained humoral response after the application of the third vaccine dose, with superior durability in comparison to the antibody kinetics after the administration of the second dose of the vaccination. These data allow for a cautiously optimistic outlook on the longevity of the antibody response after three doses of the SARS-CoV-2 vaccination in the group of LT recipients, and could help to estimate the optimal time for additional booster vaccinations.

## Figures and Tables

**Table 1:** Patient characteristics are presented as absolute number *n* and percentage or as median and interquartile range.

Patient Characteristics	LT Recipients *n*/[%]	HCWs *n*/[%]	*p*-Value
Total patient number, *n* [%]	300	122	-
Sex (male/female), *n* [%]	167 [55.7]/133 [44.3]	70 [57.4]/52 [42.6]	0.829
Age at basic immunization (years), median [IQR]	56.5 [48–64]	57 [51–61]	0.505
Age of recipient at LT, median [IQR]	47 [37–55]	-	-
Time between first and second vaccinations, median [IQR]	42 [35–42]	46 [44–48]	<0.001
Time between second and booster vaccinations (days), median [IQR]	187 [174–198]	189 [185–195]	0.020

LT: liver transplantation; HCWs: healthcare workers; IQR: interquartile range.

**Table 2:** Indications for liver transplantation are presented as absolute number *n* and percentage.

Indication for LT	*n* [%]
Alcohol-induced liver cirrhosis	45 [15]
Hepatocellular carcinoma	42 [14]
Autoimmune hepatitis	40 [13]
Primary sclerosing cholangitis	19 [6]
Acute liver failure	18 [6]
Hepatitis C virus	17 [5.7]
NASH	16 [5.3]
Cryptogenic liver cirrhosis	15 [5]
Budd–Chiari syndrome	11 [3.7]
Hepatitis B/D virus	8 [2.7]
Bile duct atresia	8 [2.7]
Cystic liver	8 [2.7]
Hepatitis B virus	6 [2]
Wilson’s disease	6 [2]
Liver cirrhosis	5 [1.7]
Primary biliary cholangitis	4 [1]
Others ^1^	32 [10.7]

Others ^1^: α-1 antitrypsin deficiency, secondary sclerosing cholangitis, cystic fibrosis, hemochromatosis, intrahepatic cholestasis syndrome, and Caroli syndrome.

**Table 3:** Comparison of LT patients who had positive and negative SARS-CoV-2 serology after second immunization. Patient characteristics are given as absolute number *n* and percentage or as median and interquartile range (IQR).

Patient Characteristics	LT Recipients SARS-CoV-2 IgG Positive	LT Recipients SARS-CoV-2 IgG Negative	*p*-Value
Total patient number, *n* [%]	158/213 [74]	55/213 [26]	-
Sex (male/female), *n* [%]	91 [57.6]/67 [42.4]	26 [47.3]/29 [52.7]	0.210
Age at basic immunization (years), median [IQR]	55.5 [41–63]	58 [51–66]	0.015
Time between first and second vaccinations, median [IQR]	42 [35–42]	42 [37–42]	0.363
**Immunosuppressive Therapy**	**Median [IQR]**	**Median [IQR]**	***p*-Value**
Prednisolone (*n* = 16) No prednisolone (*n* = 197)	9 [6] 149 [94]	7 [13] 48 [87]	0.133
MMF (*n* = 64) No MMF (*n* = 149)	29 [18] 129 [82]	35 [64] 20 [36]	0.001
Tacrolimus-based immunosuppression (*n* = 191) No tacrolimus (*n* = 22)	141 [89] 17 [11]	50 [91] 5 [9]	1.000
Everolimus (*n* = 9) No everolimus (*n* = 204)	6 [4] 152 [96]	3 [6] 52 [94]	0.698

LT: liver transplantation; IQR: interquartile range; MMF: mycophenolate mofetil; SARS-CoV-2: severe acute respiratory syndrome coronavirus type 2; IgG: immunoglobulin G.

## Data Availability

The data that support the findings of this study are available from the corresponding author upon reasonable request.
